# COVID-19 and Liver Injury: A Systematic Review and Meta-Analysis

**DOI:** 10.7759/cureus.9424

**Published:** 2020-07-27

**Authors:** Jawad Ahmed, Tehlil Rizwan, Farheen Malik, Raniyah Akhter, Mehreen Malik, Junaid Ahmad, Abdul Wasay Khan, Muhammad A Chaudhary, Muhammad Shariq Usman

**Affiliations:** 1 Internal Medicine, Dow University of Health Sciences, Karachi, PAK; 2 Pulmonology, Fazaia Ruth Phau Medical College, Pakistan Air Force (PAF) Hospital, Karachi, PAK; 3 Anesthesiology, Aga Khan University, Karachi, PAK; 4 Internal Medicine, Liaquat University of Medical and Health Sciences, Jamshoro, PAK; 5 Pediatrics, University of Kansas School of Medicine - Wichita, Wichita, USA; 6 Family Medicine, WellSpan Good Samaritan Hospital, Lebanon, USA; 7 Center for Surgery and Public Health, Harvard Medical School/Harvard T. H. Chan School of Public Health, Boston, USA; 8 Internal Medicine, Civil Hospital Karachi, Dow University of Health Sciences, Karachi, PAK

**Keywords:** liver injury, liver abnormalities, coronavirus disease 2019, covid-19, liver injury biomarkers, liver enzymes, hepatic injury, sars-cov-2

## Abstract

Background and Aims

The prevalence and extent of liver damage in coronavirus disease 2019 (COVID-19) patients remain poorly understood, primarily due to small-sized epidemiological studies with varying definitions of “liver injury”. We conducted a meta-analysis to derive generalizable, well-powered estimates of liver injury prevalence in COVID-19 patients. We also aimed to assess whether liver injury prevalence is significantly greater than the baseline prevalence of chronic liver disease (CLD). Our secondary aim was to study whether the degree of liver injury was associated with the severity of COVID-19.

Materials and Methods

Electronic databases (PubMed and Scopus) were systematically searched in June 2020 for studies reporting the prevalence of baseline CLD and current liver injury in hospitalized COVID-19 patients. Liver injury was defined as an elevation in transaminases >3 times above the upper limit of normal. For the secondary analysis, all studies reporting mean liver enzyme levels in severe versus non-severe COVID-19 patients were included. A random-effects model was used for meta-analysis. Proportions were subjected to arcsine transformation and pooled to derive pooled proportions and corresponding 95% confidence intervals (CIs). Subgroup differences were tested for using the chi-square test and associated p-value. Means and their standard errors were pooled to derive weighted mean differences (WMDs) and corresponding 95% CIs.

Results

Electronic search yielded a total of 521 articles. After removal of duplicates and reviewing the full-texts of potential studies, a total of 27 studies met the inclusion criteria. Among a cohort of 8,817 patients, the prevalence of current liver injury was 15.7% (9.5%-23.0%), and this was significantly higher than the proportion of patients with a history of CLD (4.9% [2.2%-8.6%]; p < 0.001). A total of 2,900 patients in our population had severe COVID-19, and 7,184 patients had non-severe COVID-19. Serum ALT (WMD: 7.19 [4.90, 9.48]; p < 0.001; I^2 ^= 69%), AST (WMD: 9.02 [6.89, 11.15]; p < 0.001; I^2 ^= 73%) and bilirubin levels (WMD: 1.78 [0.86, 2.70]; p < 0.001; I^2 ^= 82%) were significantly higher in patients with severe COVID-19 when compared to patients with non-severe disease. Albumin levels were significantly lower in patients with severe COVID-19 (WMD: -4.16 [-5.97, -2.35]; p < 0.001; I^2 ^= 95%).

Conclusions

Patients with COVID-19 have a higher than expected prevalence of liver injury, and the extent of the injury is associated with the severity of the disease. Further studies are required to assess whether hepatic damage is caused by the virus, medications, or both.

## Introduction

Severe acute respiratory syndrome coronavirus 2 (SARS-Cov-2), the virus responsible for coronavirus disease 2019 (COVID-19), mainly affects the respiratory system causing symptoms of fever, fatigue, cough, dyspnea, loss of appetite, muscle and joint pains [1]. However, the incidence of vomiting, nausea, and diarrhea have also been reported, suggesting the involvement of gastrointestinal and hepatobiliary systems [1,2]. SARS-Cov-2 enters cells through the angiotensin-converting enzyme-2 (ACE2) protein. Apart from type II alveolar epithelial cells of the lung, ACE2 protein is also expressed in the bile ducts cells [3]. This suggests that SARS-Cov-2 could potentially infect bile duct cells and cause abnormal liver function tests. According to one recent study, liver biopsy specimens of COVID-19 patients demonstrated moderate microvascular steatosis and mild lobular and portal activity, suggesting liver injury [4].

Although, in theory, liver injury in COVID-19 patients is possible, the actual prevalence and extent of liver damage in these patients remain poorly understood. This is primarily because most published COVID-19 studies are small-sized, often lack adjustment for baseline chronic liver disease (CLD), and have inconsistent definitions of “liver injury”. In this meta-analysis, we aim to provide well-powered and generalizable estimates of the prevalence of liver injury in COVID-19 patients while making sure to keep the definition of “liver injury” consistent. We also seek to assess whether the prevalence of liver injury in COVID-19 patients is significantly different from the prevalence of baseline CLD in these patients. A secondary aim of this study is to assess for any significant differences in serum biomarkers of liver injury (alanine transaminase [ALT], aspartate transaminase [AST], total bilirubin, and albumin) in patients with severe versus non-severe COVID-19.

## Materials and methods

This meta-analysis was conducted according to the Preferred Reporting Items for Systemic Reviews and Meta-Analyses (PRISMA) guidelines [5].

Literature search

PubMed and Scopus were searched from the inception of databases till June 18, 2020, using the following search string: (“novel coronavirus” OR “2019‐nCoV” OR “severe acute respiratory syndrome coronavirus 2” OR “SARS-CoV-2” OR “coronavirus disease 2019” OR “COVID-19”) AND (“Aspartate Aminotransferases” OR “SGOT” OR “Alanine Transaminase” OR “Alanine aminotransferase” OR “SGPT” OR “Albumin” OR “Bilirubin” OR “Liver”) AND (“hepatic injury” OR “liver injury” OR “liver damage” OR “liver abnormality”). Google Scholar was also searched for grey literature. No language and time restrictions were set. The search strategy for both databases is shown in Table 1.

**Table 1:** Search strategy for electronic databases

Electronic database	Search strategy
PubMed	(((((("novel coronavirus"[All Fields] OR "2019-nCoV"[All Fields]) OR "severe acute respiratory syndrome coronavirus 2"[All Fields]) OR "SARS-CoV-2"[All Fields]) OR "coronavirus disease 2019"[All Fields]) OR "COVID-19"[All Fields]) AND ((((((("Aspartate Aminotransferases"[All Fields] OR "SGOT"[All Fields]) OR "Alanine Transaminase"[All Fields]) OR "Alanine aminotransferase"[All Fields]) OR "SGPT"[All Fields]) OR "Albumin"[All Fields]) OR "Bilirubin"[All Fields]) OR "Liver"[All Fields])) AND ((("hepatic injury"[All Fields] OR "liver injury"[All Fields]) OR "liver damage"[All Fields]) OR "liver abnormality"[All Fields])
Scopus	( TITLE-ABS-KEY ( "liver injury" OR " liver failure" OR "hepatic damage" OR "liver function abnormality" OR "hepatic abnormality") AND TITLE-ABS-KEY ( "ALT" OR " alanine transaminase" OR "SGPT" ) AND TITLE-ABS-KEY ( "AST" OR " Aspartate transaminase" OR "SGOT" ) AND TITLE-ABS-KEY ( "bilirubin" OR " total bilirubin") AND TITLE-ABS-KEY ( "albumin" OR "serum albumin") AND TITLE-ABS-KEY ( "COVID-19" OR "SARS-CoV-2" OR "coronavirus disease" ))

Study selection

All the articles were exported to EndNote Reference Library version X4 (Clarivate Analytics, Philadelphia, PA) for screening and removal of duplicates. Studies were narrowed down based on titles and abstracts, and final inclusion was performed after reviewing the full texts of articles. Studies were selected independently by two reviewers, and a third reviewer resolved any conflict regarding inclusion.

Inclusion criteria and definitions

We included all studies among COVID-19 patients that defined liver injury as an elevation in transaminases >3 times above the upper limit of normal (ULN). To study whether the extent of liver injury was associated with severity of COVID-19, all studies that reported serum ALT, AST, total bilirubin, or albumin levels in severe versus non-severe COVID-19 patients were included. Most of the studies assessed COVID-19 severity according to either the World Health Organization interim guidance for COVID-19 or the guidelines for the diagnosis and management of COVID-19 by the National Health Commission of China, thus ensuring minimum heterogeneity in severity criteria. Case reports and studies that did not report the definition of liver injury were excluded.

Data extraction

Data extraction was performed independently by two reviewers, and, in case of any conflict, the opinion of a third reviewer was sought. Characteristics of included studies, patients’ baseline information, liver injury biomarkers, and criteria for liver injury were extracted from the studies on a predesigned form.

Statistical analysis

Open MetaAnalyst and Review Manager Version 5.4 were used for all statistical analyses. Proportions from studies were subjected to arcsine transformation and pooled using a random-effects model to derive the pooled proportions and corresponding 95% confidence intervals (CIs). The chi-square test was used to assess for any significant differences between baseline CLD prevalence and prevalence of liver injury. Continuous variables were also pooled using a random-effects model to derive the weighted mean difference (WMD) and 95% CIs. The Higgins I2 statistic was used to evaluate heterogeneity and a value of 25%-50% was considered mild, 50%-75% as moderate, and >75% as severe heterogeneity. Publication bias was assessed through visual inspection of the funnel plot. A p-value of less than 0.05 was considered significant in all cases.

## Results

**Figure 1:**
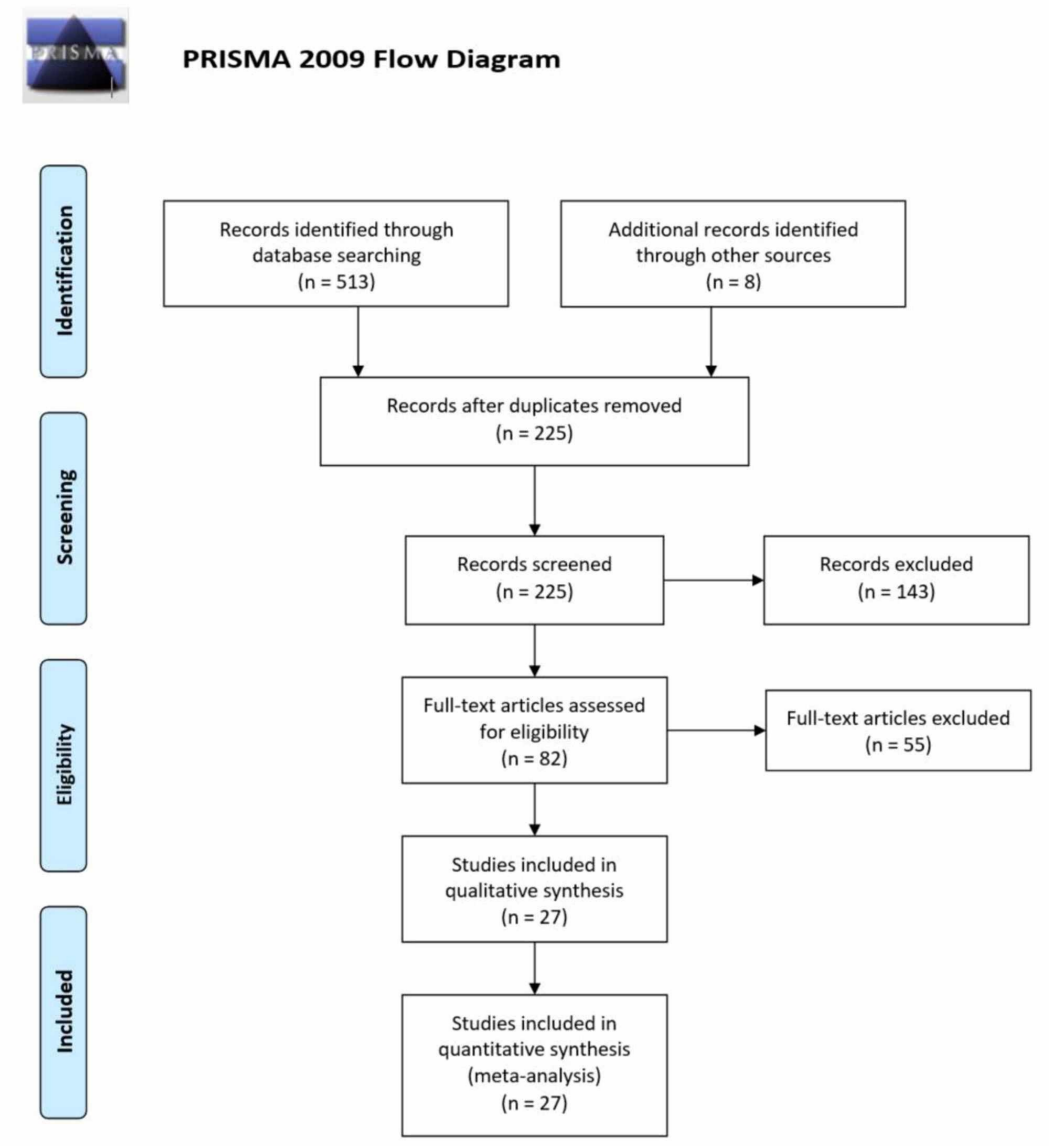
PRISMA flow chart summarizing the literature search PRISMA, Preferred Reporting Items for Systemic Reviews and Meta-Analyses

**Figure 2:**
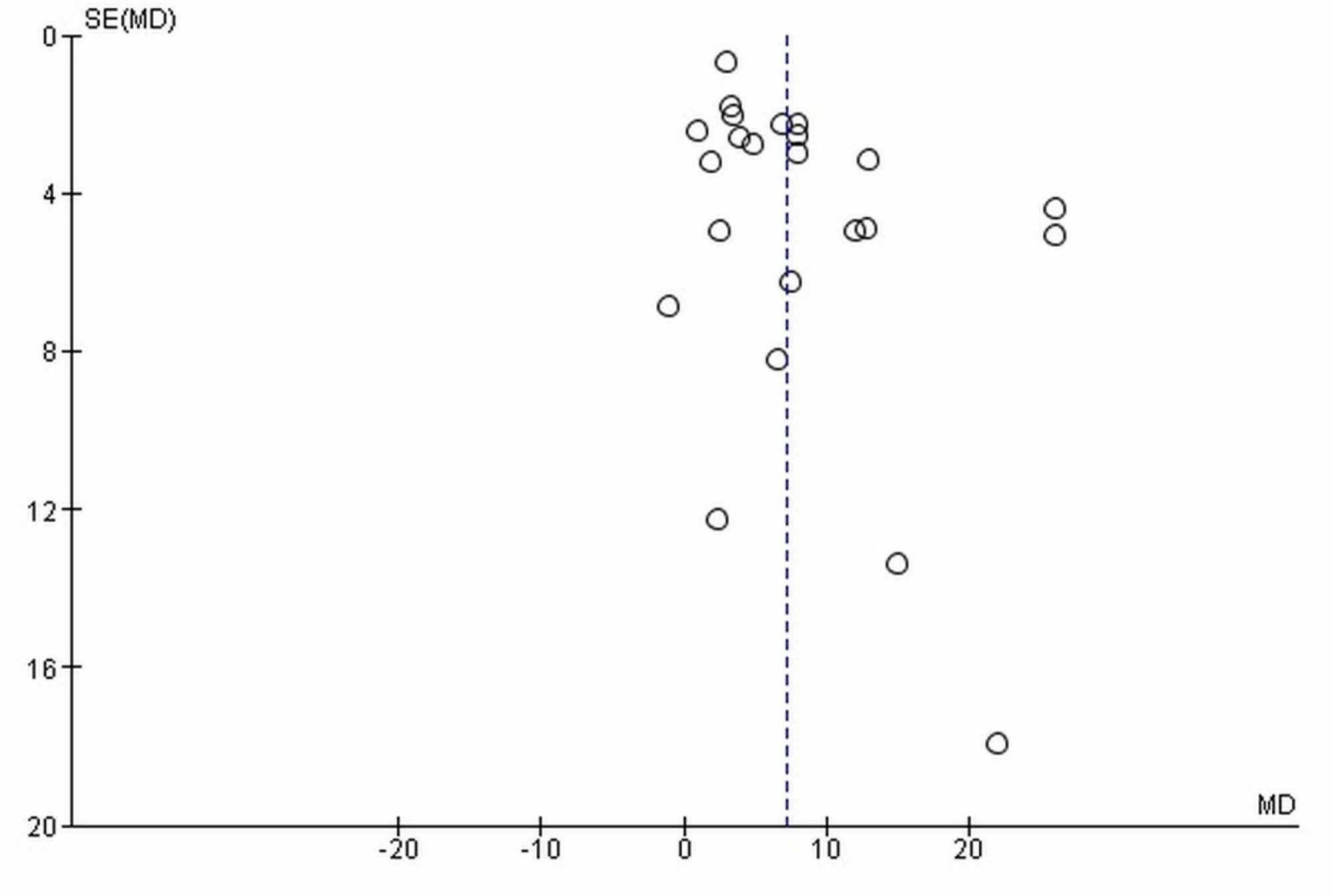
Funnel plot for publication bias The funnel plot is based on serum ALT levels. SE, standard error; MD, mean difference; ALT, alanine transaminase

The initial search yielded 513 potential articles, and eight records were identified through references of relevant studies. After exclusions, 27 studies were used in our quantitative analysis [1,2,4,6-29]. The PRISMA flowchart (Figure 1) summaries the results of our literature search. The baseline characteristics and outcomes of included studies are given in Table 2. Visual inspection of the funnel plot (based on serum ALT outcome) showed no publication bias (Figure 2).

**Table 2:** Baseline characteristics and demographics of the included studies CLD, chronic liver disease; ALT, alanine transaminase; AST, aspartate transaminase; T. bilirubin, total bilirubin; NR, not reported

Author name	Location	Study design	Total	Males (%)	Age (years)	Baseline CLD (%)	Outcomes
Bloom et al. [2]	USA	Prospective	60	39 (65.0)	57	4 (7.0)	Liver injury
Cai et al. [6]	China	Retrospective	298	145 (48.7)	47.5	28 (9.4)	Liver injury; biomarkers: ALT, AST, and T. bilirubin
Cai et al. [7]	China	Cross-sectional	417	298 (71.5)	47.32	21 (5.04)	Liver injury; biomarkers: ALT, AST, and T. bilirubin
Chen et al. [8]	China	Retrospective	21	17 (81.0)	56.5	NR	Biomarkers: ALT, AST, T. bilirubin, and albumin
Chen et al. [4]	China	Retrospective	274	171 (62.0)	59.5	NR	Biomarkers: ALT, AST, T. bilirubin, and albumin
Deng et al. [9]	China	Retrospective	225	124 (55.1)	54.5	NR	Biomarkers: ALT and AST
Fu et al. [10]	China	Retrospective	355	190 (53.5)	>60=115	9 (2.5)	Biomarkers: ALT, AST, T. bilirubin, and albumin
Gao et al. [11]	China	Retrospective	43	26 (60.5)	44.08	NR	Biomarkers: ALT and AST
Huang C et al. [1]	China	Retrospective	41	30 (73.0)	49	1 (3.6)	Biomarkers: ALT and AST
Jin et al. [12]	China	Retrospective	651	331 (50.8)	45.61	25 (3.8)	Biomarkers: ALT, AST, T. bilirubin, and albumin
Lei et al. [13]	China	Retrospective	5,771	2,724 (47.2)	56	81 (1.4)	Liver injury
Liu et al. [14]	China	Retrospective	78	39 (50.0)	51.5	NR	Biomarkers: ALT, AST, and albumin
Mo et al. [15]	China	Retrospective	155	86 (55.5)	53.5	7 (4.5)	Biomarkers: ALT, AST, and albumin
Pan et al. [16]	China	Retrospective	204	107 (52.5)	52.9	2 (0.01)	Biomarkers: ALT, AST, T. bilirubin, and albumin
Phipps et al. [17]	USA	Retrospective	2,273	1,297 (57.1)	65	114 (5.0)	Liver injury
Qian et al. [18]	China	Retrospective	91	37 (40.7)	57.5	NR	Biomarkers: ALT, AST, and albumin
Qu et al. [19]	China	Retrospective	30	16 (53.3)	54.7	Excluded (0)	Biomarkers: ALT and AST
Ruan et al. [20]	China	Retrospective	150	102 (68)	58.5	4 (2.7)	Biomarkers: T. bilirubin and albumin
Wan et al. [21]	China	Retrospective	135	72 (53.3)	50	2 (1.5)	Biomarkers: ALT, AST, T. bilirubin, and albumin
Wang et al. [22]	China	Retrospective	138	75 (54.3)	58.5	4 (2.9)	Biomarkers: ALT, AST, and T. bilirubin
Wang et al. [23]	China	Retrospective	69	32 (46)	53.7	1 (1.4)	Biomarkers: ALT and AST
Wu et al. [24]	China	Retrospective	201	128 (63.7)	53.25	7 (3.5)	Biomarkers: ALT, AST, T. bilirubin, and albumin
Xie et al. [25]	China	Retrospective	79	44 (55.7)	60	Excluded (0)	Biomarkers: ALT, AST, and T. bilirubin
Yang et al. [26]	China	Retrospective	52	35 (67.0)	58.25	16 (30.8)	Biomarkers: T. bilirubin
Zhang et al. [27]	China	Retrospective	645	328 (50.9)	40.77	25 (3.9)	Biomarkers: ALT, AST, T. bilirubin, and albumin
Zhou et al. [28]	China	Retrospective	34	17 (50.0)	65	NR	Biomarkers: ALT and AST
Zhou et al. [29]	China	Retrospective	191	119 (62.0)	60.5	NR	Biomarkers: ALT and albumin

Liver injury

**Figure 3:**
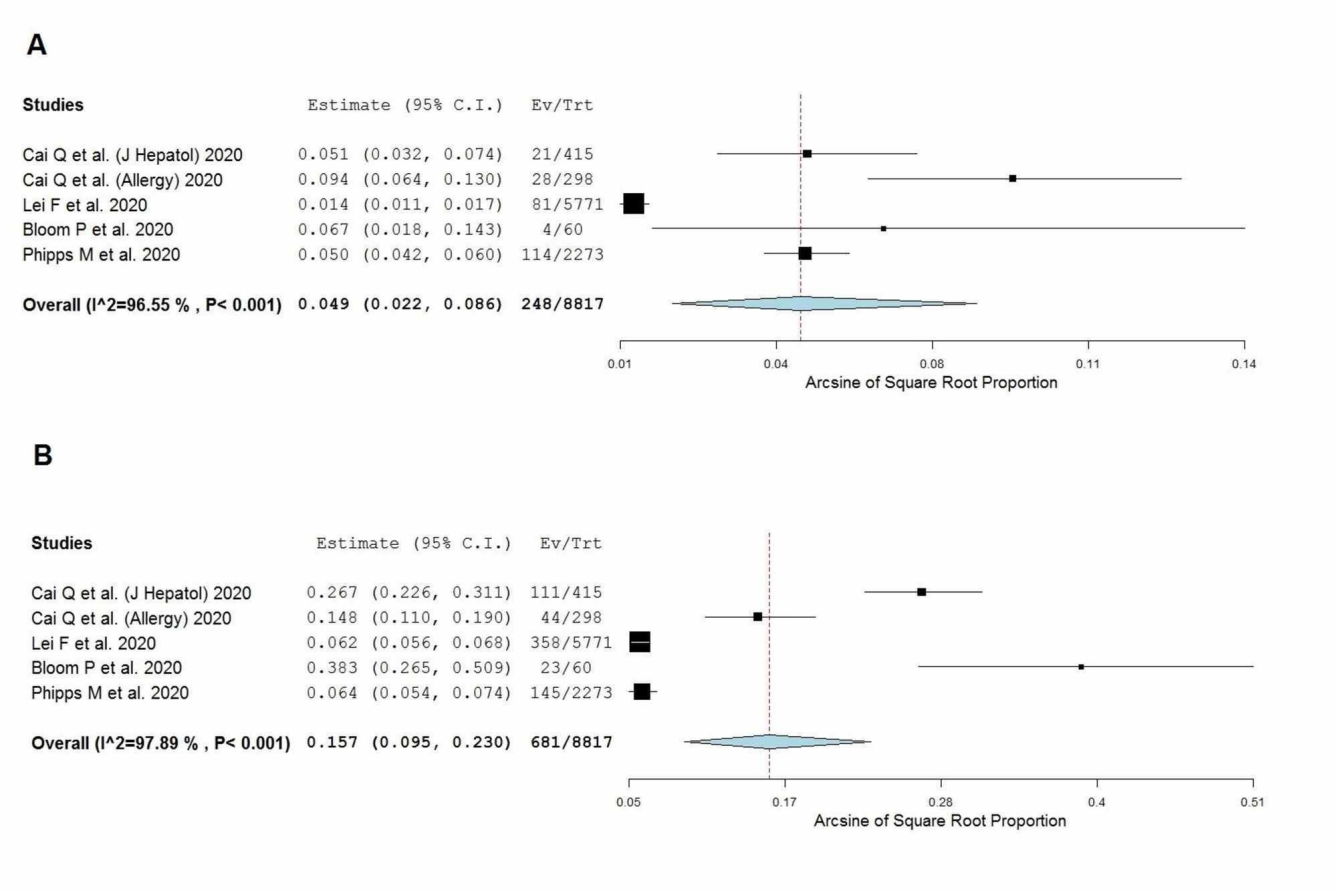
Pooled prevalence of (A) patients with a history of chronic liver disease and (B) patients with liver injury CI, confidence interval; Ev/Trt, events/total; I^2^, heterogeneity

Liver injury was defined as an elevation in transaminases >3 times above the ULN. Five studies, including 8,817 COVID-19 positive patients, met the pre-defined inclusion criteria for liver injury [2,6,7,13,17]. The pooled proportion of patients with a history of CLD was 4.9% (2.2%-8.6%]) The prevalence of liver injury was 15.7% (9.5%-23.0%), as shown in Figure 3, and this was significantly higher than the baseline CLD prevalence (p < 0.001).

Biomarkers of liver injury in severe versus non-severe COVID-19 patients

**Figure 4:**
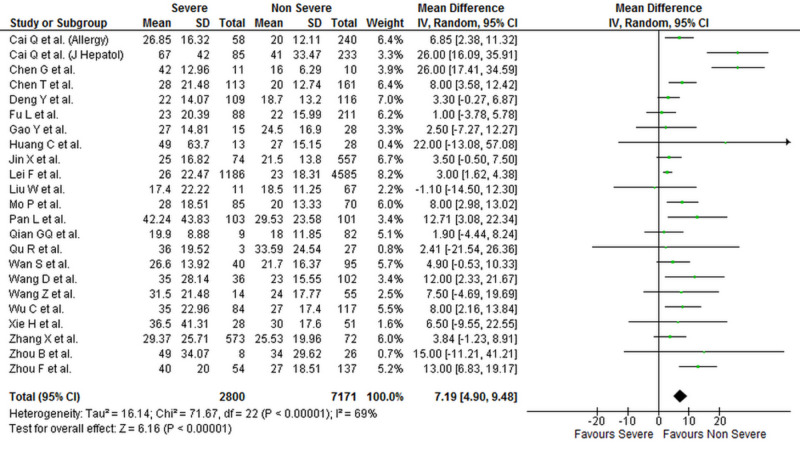
Forest plot showing the association between serum ALT levels and severity of disease in COVID-19 patients SD, standard deviation; IV, inverse variance; CI, confidence interval; ALT, alanine transaminase; COVID-19, coronavirus disease 2019

Levels of liver biomarkers were reported by 25 studies comprising 10,084 patients (2,900 severe COVID-19 and 7,184 non-severe COVID-19 patients) [1,4,6-16,18-29]. Our analysis shows that serum ALT (WMD: 7.19 [4.90, 9.48]; p < 0.001; I^2^ = 69%), AST (WMD: 9.02 [6.89, 11.15]; p < 0.001; I^2^ = 73%), and total bilirubin levels (WMD: 1.78 [0.86, 2.70]; p < 0.001; I^2^ = 82%) were significantly higher in patients with severe COVID-19 when compared to patients with non-severe disease. Albumin levels were significantly lower in patients with severe COVID-19 (WMD: -4.16 [-5.97, -2.35]; p < 0.001; I^2^ = 95%). The individual forest plots for ALT, AST, total bilirubin, and albumin are shown in Figures 4-­7, respectively.

**Figure 5:**
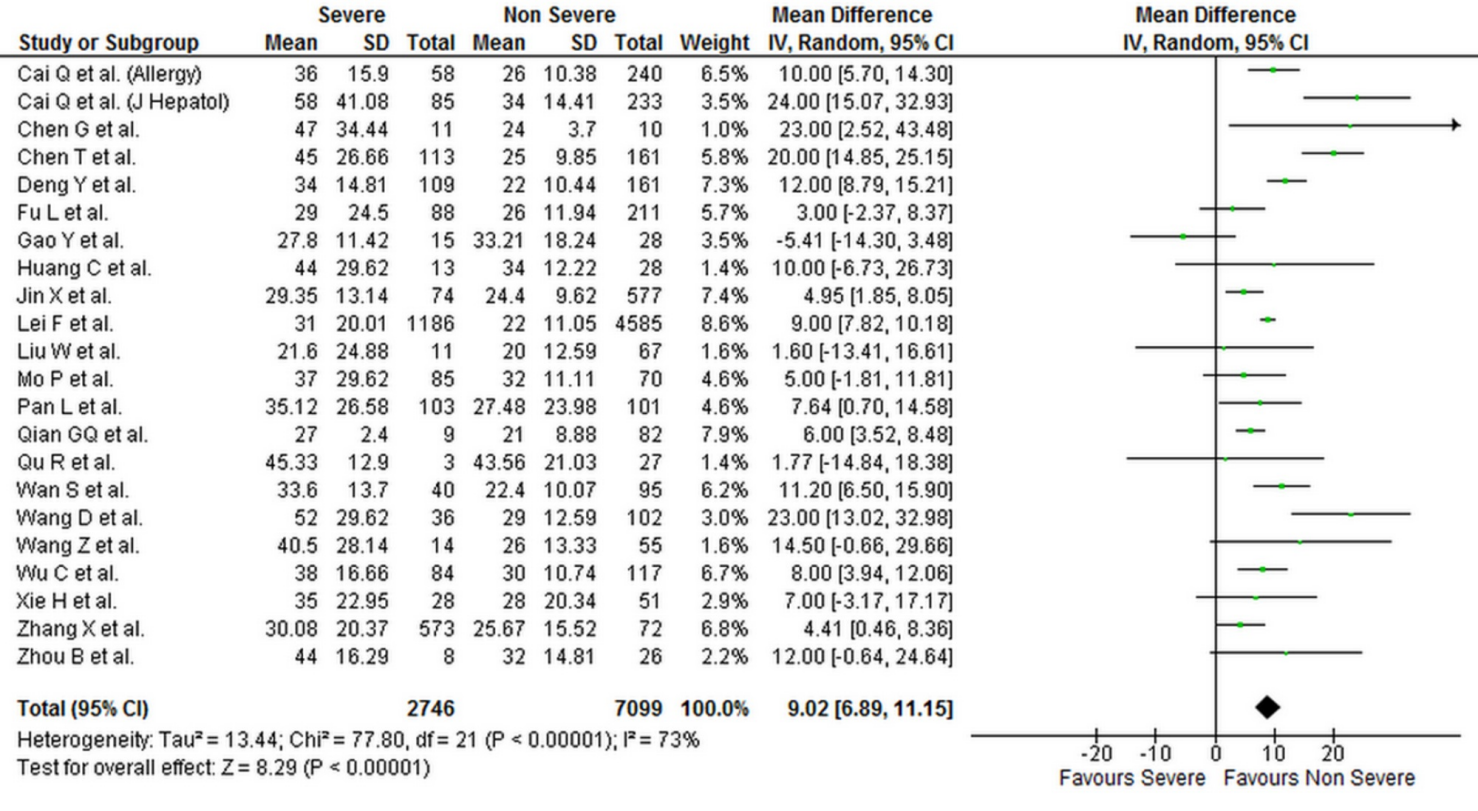
Forest plot showing the association between serum AST levels and severity of disease in COVID-19 patients SD, standard deviation; IV, inverse variance; CI, confidence interval; AST, aspartate transaminase; COVID-19, coronavirus disease 2019

**Figure 6:**
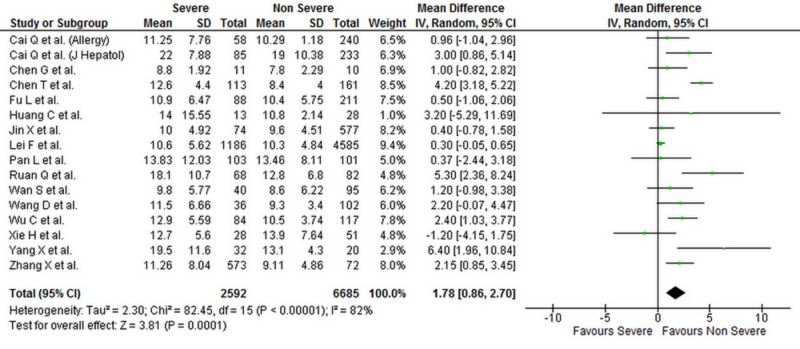
Forest plot showing the association between serum total bilirubin levels and severity of disease in COVID-19 patients SD, standard deviation; IV, inverse variance; CI, confidence interval; COVID-19, coronavirus disease 2019

**Figure 7:**
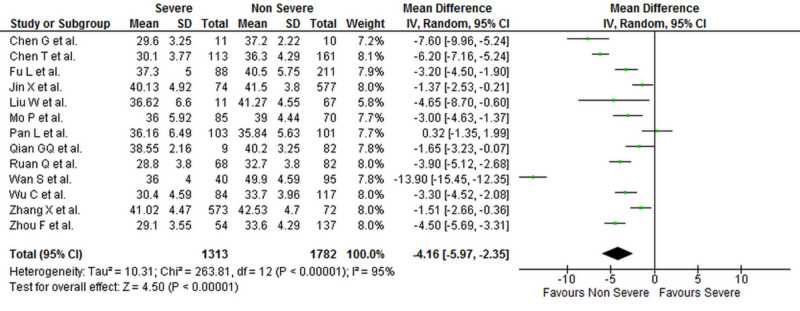
Forest plot showing the association between serum albumin levels and severity of disease in COVID-19 patients SD, standard deviation; IV, inverse variance; CI, confidence interval; COVID-19, coronavirus disease 2019

The serum levels of all biomarkers for severe and non-severe COVID-19 patients in each study are given in Table 3. The criterion used to diagnose COVID-19 in each study is given in the Appendices section.

**Table 3:** Serum levels of ALT, AST, total bilirubin, and albumin among severe and non-severe COVID-19 patients in the included studies COVID-19, coronavirus disease 2019; ALT, alanine transaminase; AST, aspartate transaminase

Author Name	No. of COVID-19 patients		Mean serum ALT levels		Mean serum AST levels		Mean serum total bilirubin levels		Mean serum albumin levels	
	Severe COVID-19	Non-severe COVID-19	Severe COVID-19	Non-severe COVID-19	Severe COVID-19	Non-severe COVID-19	Severe COVID-19	Non-severe COVID-19	Severe COVID-19	Non-severe COVID-19
Cai et al. [6]	58	240	26.85±16.32	20±12.11	36±15.9	26±10.83	11.25±7.76	10.9±1.18	-	-
Cai et al. [7]	85	233	67±42.0	41±33.47	58±41.08	34±14.41	22±7.88	19±10.38	-	-
Chen et al. [8]	11	10	42±12.96	16±6.29	47±34.44	24±3.70	8.80±1.92	7.80±2.29	29.60±3.25	37.20±2.22
Chen et al. [4]	113	161	28.00±21.48	20.00±12.74	45.00±26.66	25.00±9.85	12.60±4.4	8.40±4.00	30.10±3.77	36.30±4.29
Deng et al. [9]	109	116	22.00±14.07	18.70±13.2	34.00±14.81	22.00±10.44	-	-	-	-
Fu et al. [10]	88	211	23.0±20.39	22.0±15.99	29.0±24.5	26.0±11.94	10.9±6.47	10.4±5.75	37.3±5.0	40.5±5.75
Gao et al. [11]	15	28	27.00±14.81	24.50±16.29	27.80±11.42	33.21±18.24	-	-	-	-
Huang et al. [1]	13	28	49.00±63.7	27.00±15.18	44.00±29.62	34.00±12.22	14.00±15.55	10.80±2.14	-	-
Jin et al. [12]	74	577	25.00±16.82	21.50±13.18	29.35±13.14	24.40±9.62	10.00±4.92	9.60±4.51	40.13±4.92	41.50±3.8
Lei et al. [13]	1186	4585	26.0±22.47	23.0±18.31	31.0±20.01	22.0±11.05	10.6±5.62	10.3±4.84	-	-
Liu et al. [14]	11	67	17.40±22.22	18.50±11.25	21.60±24.88	20.00±12.59	-	-	36.62±6.6	41.27±4.55
Mo et al. [15]	85	70	28.00±18.51	20.00±13.33	37.00±29.62	32.00±11.11	-	-	36.00±5.92	39.00±4.44
Pan et al. [16]	103	101	42.24±43.83	29.53±23.58	35.12±26.58	27.48±23.98	13.83±12.03	13.46±8.11	36.16±6.49	35.84±5.63
Qian et al. [18]	9	82	19.90±8.88	18.00±11.85	27.00±2.4	21.00±8.88	-	-	38.55±2.16	40.20±3.25
Qu et al. [19]	3	27	36.00±19.52	33.59±24.54	45.33±12.9	43.56±21.03	-	-	-	-
Ruan et al. [20]	68	82	-	-	-	-	18.10±10.7	12.80±6.8	28.80±3.8	32.70±3.8
Wan et al. [21]	40	95	26.60±13.92	21.70±16.37	33.60±13.70	22.40±10.07	9.80±5.77	8.60±6.22	36.00±4.0	49.90±4.59
Wang et al. [22]	36	102	35.00±28.14	23.00±15.55	52.00±29.62	29.00±12.59	11.50±6.66	9.30±3.40	-	-
Wang et al. [23]	14	55	31.50±21.48	24.00±17.77	40.50±28.14	26.00±13.33	-	-	-	-
Wu et al. [24]	84	117	35.00±22.96	27.00±17.40	38.00±16.66	30.00±10.74	12.90±5.59	10.50±3.74	30.40±4.59	33.70±3.96
Xie et al. [25]	28	51	36.5±41.31	30.0±17.6	35±22.95	28±20.34	12.7±5.6	13.9±7.64	-	-
Yang et al. [26]	32	20	-	-	-	-	19.50±11.6	13.10±4.3	-	-
Zhang et al. [27]	573	72	29.37±25.71	25.53±19.96	30.08±20.37	25.67±15.52	11.26±8.04	9.11±4.86	41.02±4.47	42.53±4.7
Zhou et al. [28]	8	26	49.00±34.07	34.00±29.62	44.00±16.29	32.00±14.81	-	-	-	-
Zhou et al. [29]	54	137	40.00±20.0	27.00±18.51	-	-	-	-	29.10±3.55	33.60±4.29

## Discussion

Our meta-analysis has three salient findings. Firstly, almost 16% of the COVID-19 positive patients had a substantial elevation in enzymes (>3 times the ULN). Secondly, the prevalence of current liver injury in hospitalized COVID-19 patients was significantly higher than the prevalence of patients with known pre-COVID CLD. Thirdly, the extent of liver damage was associated with the severity of COVID-19.

Although our study demonstrates a clear association between COVID-19 and liver injury, mechanisms for liver injury remain unclear. Direct injury by SARS-CoV-2 has been proposed as a likely mechanism [3]. The expression of ACE2 protein (entry receptor for SARS-CoV-2) on bile duct cells supports the possibility of virus-mediated liver damage [3]. However, there is a lower frequency of receptors found in liver cells than bile duct cells, and trends show an elevation in aminotransferases rather than alkaline phosphate and gamma-glutamyl transferase [3,6,7,17]. Thus, alternate mechanisms must be considered as well.

Apart from direct viral-mediated injury, drug-induced liver injury must also be given consideration. Acetaminophen, a drug commonly used by COVID-19 patients, is known to cause hepatic injury at doses >7.5 to 10 g in adults [30]. In addition, the simultaneous use of multiple antiviral therapies and antibiotics in these patients can be hepatotoxic [17,21-23]. Aggressive treatment in patients with more severe disease may explain the association of liver injury with disease severity seen in our study.

Systemic effects of COVID-19 could be another possible explanation for the liver injury. It is proven that SARS-Cov-2 infects the lung causing hypoxia and, in severe cases, acute respiratory distress syndrome, sepsis, and multi-organ failure [1,4,9,17]. It can be imagined that sepsis in COVID-19 leads to hypoxic injury and ischemia of the liver, causing elevated liver biochemistries, which further explains why serum ALT, AST, and total bilirubin levels are higher in severe/ICU COVID-19 patients than non-severe patients, as demonstrated in our study [6,7,17].

To the best of our knowledge, this is the first meta-analysis to report the prevalence of liver injury in COVID-19 patients while keeping the definition of liver injury standard and accounting for baseline CLD history. Thus, these results should be generalizable across cohorts of hospitalized COVID-19 patients. Our results should stimulate further research interest in the area in order to uncover mechanisms contributing to liver injury.

Limitations

This study has certain important limitations. Firstly, the quality of data-collection methods and data reported in individual studies cannot be ascertained. Secondly, patients can sometimes be unaware of underlying CLD (e.g., in non-alcoholic fatty liver disease), and these patients may be misclassified as having no CLD. This could have led to an underestimation of baseline CLD prevalence. Third, as shown, estimates from our study had significantly high heterogeneity. This is likely due to the inclusion of primarily small studies with varied prevalence. Lastly, all included studies were of observational nature, and there is a need for randomized trials on this aspect of COVID-19. Although meta-analysis can increase the power and provide better estimates, the results are intended to offer early insight and should not be considered a replacement for large-scale observational studies that are being awaited.

## Conclusions

This systematic review and meta-analysis shows that COVID-19 is associated with an increased incidence of liver injury. Furthermore, the extent of derangement in serum liver function markers is associated with the severity of COVID-19. Future studies should adopt a pre-defined criterion for reporting liver injury and exclude those patients from the analyses that had any form of baseline derangement in liver enzymes and biomarkers at hospital admission. A standard protocol should be formed for COVID-19 patients to identify them as "with liver injury" or "without liver injury", as this will lead to uniform reporting and low bias in studies.

## Appendices

**Table 4:** Diagnostic criteria used for the assessment of severity of COVID-19 among patients in the included studies WHO, World Health Organization; COVID-19, coronavirus disease 2019; 2019-nCoV, 2019 novel coronavirus

Author name	Diagnostic criteria	
		Cai et al. [6]	WHO interim guidance for COVID-19	
Cai et al. [7]	Handbook of Prevention and Treatment of the Pneumonia Caused by the 2019-nCoV by National Health Commission of China	
Chen et al. [8]	6th edition guidelines for diagnosis and management of COVID-19 by the National Health Commission of China	
Chen et al. [4]	6th edition guidelines for diagnosis and management of COVID-19 by the National Health Commission of China	
Deng et al. [9]	6th edition guidelines for diagnosis and management of COVID-19 by the National Health Commission of China	
Fu et al. [10]	Diagnosed on basis of typical clinical manifestations accompanied with characteristic chest radiology changes	
Gao et al. [11]	WHO interim guidance for COVID-19	
Huang et al. [1]	Diagnosis of pneumonia was based on clinical characteristics, chest imaging, and excluding common pathogens that cause pneumonia	
Jin et al. [12]	6th edition guidelines for diagnosis and management of COVID-19 by the National Health Commission of China	
Lei et al. [13]	WHO interim guidance for COVID-19	
Liu et al. [14]	4th edition guidelines for diagnosis and management of COVID-19 by the National Health Commission of China	
Mo et al. [15]	Diagnosis of pneumonia was based on clinical characteristics and chest imaging	
Pan et al. [16]	WHO interim guidance for COVID-19	
Qian et al. [18]	4th and 5th edition guidelines for diagnosis and management of COVID-19 by the National Health Commission of China	
Qu et al. [19]	6th edition guidelines for diagnosis and management of COVID-19 by the National Health Commission of China	
Ruan et al. [20]	Pneumonia was diagnosed on clinical characteristics and chest imaging	
Wan et al. [21]	WHO interim guidance for COVID-19	
Wang et al. [22]	WHO interim guidance for COVID-19	
Wang et al. [23]	6th edition guidelines for diagnosis and management of COVID-19 by the National Health Commission of China	
Wu et al. [24]	WHO interim guidance for COVID-19	
Xie et al. [25]	WHO interim guidance with laboratory-identified COVID-19	
Yang et al. [26]	WHO interim guidance for COVID-19	
Zhang et al. [27]	WHO interim guidance for COVID-19 and 6th edition guidelines for diagnosis and management of COVID-19 by the National Health Commission of China	
Zhou et al. [28]	4th edition guidelines for diagnosis and management of COVID-19 by the National Health Commission of China	
Zhou et al. [29]	WHO interim guidance for COVID-19 and 6th edition guidelines for diagnosis and management of COVID-19 by the National Health Commission of China	
